# ATP bioluminescence assay for evaluating cleaning practices in operating theatres: applicability and limitations

**DOI:** 10.1186/s12879-018-3505-y

**Published:** 2018-11-19

**Authors:** Tiziana Sanna, Laura Dallolio, Alessandra Raggi, Magda Mazzetti, Giovanni Lorusso, Angela Zanni, Patrizia Farruggia, Erica Leoni

**Affiliations:** 1 Department of Biomedical and Neuromotor Sciences, Unit of Hygiene, Public Health and Medical Statistics, University of Bologna, via S. Giacomo 12, 40126 Bologna, Italy; 2 Unit of Hygiene, Control of Healthcare Associated Infections, Bologna Local Health Authority, “Bellaria” Hospital, via Altura 3, 40139 Bologna, Italy; 3 Unit of Hygiene and Quality of Residencial Services, Bologna Local Health Authority, “Bellaria” Hospital, via Altura 3, 40139 Bologna, Italy

**Keywords:** Operating theatres, Surgery cleaning procedures., Microbiological contamination., ATP-bioluminescence assay

## Abstract

**Background:**

Environmental cleaning practice plays an important role in reducing microbial contamination in hospital surfaces and contributes to prevent Healthcare Associated Infections. Adenosine Triphosphate (ATP) bioluminescence assay is a commonly used method for assessing environmental cleanliness on healthcare surfaces. This study tested the feasibility of using ATP-bioluminescence assay for evaluating the efficiency of cleaning procedures in the operating theatre settings, comparing the ATP-bioluminescence test with the traditional culture method.

**Methods:**

The surfaces of 10 operating rooms of two public hospitals (140 samples in total) were examined “at rest”, in two moments of the same daily session: before the first scheduled operation (Pre), and before the second, after a clean environment was re-established (Post). Surface contamination was assessed using the cultural method to detect Total Viable Counts (TVC36°C) and ATP-bioluminescence assay (RLU).

**Results:**

The examined surfaces presented very low TVCs (geometric means: 1.8 CFU/plate; IC95%: 1.6–2.0), always compliant with the relative reference standards. No statistical correlation was found between ATP values and TVCs. However, considering the results in terms of general evaluation of hygienic quality of surfaces, the two methods were consistent in identifying the most contaminated areas (Hospital A > Hospital B; Pre > Post; most contaminated surfaces: scialytic lamp). Furthermore, the ATP mean values showed a progressive increase from surfaces with TVC = 0 to surfaces with TVC > 15 CFU/plate.

**Conclusions:**

Although not an alternative to cultural methods, the ATP-bioluminescence-assay can be a useful tool to measure the efficiency of cleaning procedures also in environments with very low microbial counts. Each health facility should identify appropriate reference values, depending on the devices used and on the basis of the analysis of the data collected through spatial and temporal sampling series. By providing a rapid feedback, the ATP-assay helps to increase the awareness of operators and allows immediate action to be taken in critical situations.

## Background

Surgical site infections (SSIs) represent 20% of all Healthcare Associated Infections (HAIs) in Europe and the United States, where they are the second most frequent type of HAI [1, 2]. SSIs result in higher morbidity and mortality of surgical patients and greatly increase healthcare costs [3]. For many years, environmental contamination has been considered a marginal risk factor in the development of nosocomial infections. Nevertheless, more recent findings indicate that a contaminated environment plays a significant role in the transmission of microorganisms, including multidrug-resistant organisms [4–6]. Operating rooms are high-risk areas, where high hygiene standards should be constantly guaranteed. Contaminated surfaces can represent a reservoir of pathogenic microorganisms, which can be spread through the air or by contact with the healthcare personnel (e.g. their hands), increasing the risk of infections [7].

In order to restore a low level of contamination, the operating room surfaces should be thoroughly cleaned on a daily basis, following protocols/procedures built on best practices correctly applied. Repeated sanitizations during the day are needed in order to restore a low level of microbial contamination between operating sessions [2, 7]. The assessment of the degree of contamination and the monitoring of the correct implementation of surgical cleaning practices, through environmental sampling, is an important measure of prevention and control of SSIs [8, 9].

Currently, there is no consensus on the standard method for objectively measuring hospital cleanliness, and there are no official limits of contamination that can be used as standards at an international level for the microbiological screening of surfaces [8]. Total Viable Count (TVC) < 2.5–5 CFU/cm^2^ on hand-touch surfaces have been proposed as a microbiologic benchmark and < 1 CFU/cm^2^ when finding a potential pathogen. These levels have not yet been standardized for the hospital setting [10]. Moreover, hospital wards are characterized by different levels of risk depending on patient risk and type of activity: operating rooms or intensive care unit surfaces are more critical for infection risk and strict limit values should be chosen.

In Italy, in the absence of a specific regulation, the ISPESL (*Istituto Superiore di Prevenzione e Sicurezza del Lavoro*) Italian Guidelines (2009) [11] suggest referring to the limits proposed in the 1999 French Guidelines (C.CLIN, 1999) [12] and confirmed by the more recent 2016 French Guidelines (C.CLIN, 2016) [13]. Based on these regulations, the standard method to test the microbiological quality of surfaces in critical hospital environments is the search for the TVC at 36 °C, applying the RODAC (Replicate Organism Direct Agar Contact) method. The TVCs in the hand-touch surfaces of the operating rooms should not exceed 5 CFU/plate (expected value), while values > 5 and ≤ 15 CFU/plate are considered acceptable, and TVC > 15 CFU/plate indicates hygiene failure, as well as the possible detection of *Staphylococcus aureus, Enterobacteriaceae*, *Pseudomonas* spp., *Aspergillus* spp.

Traditional microbiological techniques are the most commonly used methods to evaluate hygienic quality, but they require specific skills, long execution and analysis times and are therefore unsuitable for routine monitoring. In the last decade alternative methods for assessing environmental cleanliness have been proposed, including the adenosine triphosphate (ATP) bioluminescence assay, based on the measurement of levels of ATP present on an environmental surface. Bioluminescence test exploits the chemiluminescence properties of luciferin-luciferase reagent, which reacts with any ATP residue present on a substrate, emitting light and measuring the presence of organic matter [8, 14]. First applied in the food industry, ATP-bioluminescence is easy to use, provides rapid results and has already been investigated as an objective tool to assess hospital cleanliness even in high-risk areas [15–21]. ATP is an indicator of organic material presence, rather than microbial contamination, and although some studies have found a correlation between ATP levels and TVC values [15, 22], a direct relationship remains controversial and some authors have expressed their doubts about the usefulness of bioluminescence for hygiene monitoring within healthcare settings [23]. Furthermore, this method is still poorly standardized and there are currently no benchmarks or strong evidences that would recommend its use. A review on the use of ATP-bioluminescence in healthcare environments shows that the benchmark levels range from 100 to 500 RLU/100 cm^2^, depending on the device used and the type of surface investigated [15]. An ATP level of 100 RLU/100 cm^2^ is frequently used as benchmark value also for high risk environments [15, 17]. To our knowledge, the ATP-bioluminescence assay and the microbiological method have not previously been compared in the operating theatre setting.

In light of this, the present study aimed to assess the feasibility of using the bioluminescence technique as a rapid analytical method directly in situ to verify the hygienic quality of the most critical surfaces in operating theatres and the effectiveness of sanitization procedures. This study also aimed to compare the ATP-bioluminescence test with the traditional culture method used to assess the microbiological contamination of surfaces, in order to evaluate the applicability and limitations of the ATP assay as an alternative method to the microbiological monitoring.

## Methods

### Setting and sampling

Environmental sampling was conducted at the operating blocks of two hospitals in Bologna (Emilia Romagna, Northern Italy). All the operating theatres had the same structural characteristics and applied the same written operative protocol of cleaning practices, which was previously described in more detail [24]. Two moments in the same operating session were monitored: i) Pre: before the first scheduled operation of the day; ii) Post: before the second scheduled operation of the day. Sampling was always carried out “at rest”, when no patients and medical staff were in the operating theatre and after the operating rooms had been sanitized by competent personnel specifically trained (cleaners). The operating cleaning procedures had been performed during the previous evening in the “pre” sampling, while a further clean environment had been re-established between the first and the second operation (“post” sampling), in accordance with the surgery cleaning protocol previously described [24]. Sampling was carried out after at least 15 min had passed since the end of the cleaning operations, in order to avoid possible interferences of detergents and disinfectants with the bioluminescence method.

The samples were taken from 140 surfaces, identified as “critical”, on account of their being more frequently touched [11, 25, 26]. For each sampling session, seven surfaces were monitored: medical anaesthesia trolley, nurse’s computer touch screen, operating table, vitals monitor, anaesthetist’s computer touch screen, surgical lighting, and instrument table. In all operating theatres the same criterion was adopted for the selection of the areas to be monitored, trying to sample, for the same equipment, the most frequently hand-touched sites.

Surface contamination was assessed using a cultural microbiological method and ATP-bioluminescence assay.

### Microbiological analysis

Sampling was carried out using the RODAC imprint technique (UNI EN ISO 14698-1, 2004). RODAC plates containing the agarised cultural medium (diameter: 55 mm; contact surface: 24 cm^2^) were pressed on each surface for 10 s, applying a constant pressure, for a total of 140 sampled surfaces. At the end of sampling, plates were placed in refrigerated containers and transported to the laboratory for analysis. Total Viable Count (TVC) was determined on Plate Count Agar (PCA) with neutralizer (Italian Biolife, Milan, Italy). Following 48 h incubation at 36 °C, colony forming units (CFU) on PCA were counted and sub-cultured in order to identify *Staphylococcus aureus*, *Enterobacteriaceae*, Enterococci and *Pseudomonas* spp. by morphological and biochemical features (API miniaturized biochemical tests, bioMérieux, Marcy l’Etoile, France).

### ATP-bioluminescence assay

3 M™ Clean-Trace™ Surface ATP (3 M, St. Paul, MN, USA) was used, which is a single-use test device containing a chemically impregnated reagent swab for the collection of a sample from a surface. The swab was rubbed, according to the manufacturer’s instructions, on the area to be analysed, first in one direction and then in the opposite direction. Each sample was obtained from a test area of 10 × 10 cm, immediately adjacent to that used for the RODAC sampling. The samples were immediately analysed using 3 M™ Clean-Trace™ NGi Luminometer (3 M, St. Paul, MN, USA), which measures the amount of light generated by chemical reaction, and produces a result expressed in Relative Light Units (RLUs). The intensity of the light is proportional to the amount of ATP and therefore the degree of contamination.

At first, we considered as threshold value an ATP level of 100 RLU/100 cm^2^, which is frequently used as benchmark [15, 17, 27]. In addition, as this value is not standardized for the type of environment investigated in this study, an internal target value was calculated, corresponding to the 75 percentile of the detected values. The target value + a deviation of 20% was considered as internal alert value, in accordance with the recommendations of the CDC, which suggests taking into account deviations of up to 20% from the established reference values [9].

### Statistical analysis

Statistical analyses were performed using the SPSS software, version 20 (IBM SPSS Statistics, IBM Corporation, Chicago, IL, USA). The microbiological data (CFU) and ATP-bioluminescence values (RLU) were converted into Log_10_(× + 1) to normalize the non-normal distributions. The results are presented as geometric means with confidence intervals, and as medians with relative ranges (minimum and maximum). ANOVA was used to compare the differences between means. A *P* < 0.05 was considered as statistically significant. Pearson correlation coefficient and linear regression model were used to describe the relationship between TVCs and ATP-RLU values. Correlation was considered according to the following ranges of r values: < 0.3 (weak correlation), 0.3–0.7 (moderate correlation), 0.7–1 (strong correlation).

## Results

### Microbiological quality of surfaces

Using the Italian ISPESL guidelines as reference (2009), the microbial contamination of the surfaces was within the range for the expected values (≤ 5 CFU/RODAC plate) in 93.6% of samples and was acceptable (5–15 CFU/plate) in 4.3%. The TVC exceeded 15 CFU/plate only in 3 samples (2.1%) collected from 3 different operating rooms. *S. aureus, Enterobacteriaceae,* Enterococci*, Pseudomonas* spp. were never detected in any of the 140 samples*.*

### ATP-bioluminescence quality of surfaces in comparison with microbial contamination

Table 1 shows the mean RLU values in the different classes of microbial contamination identified on the basis of the RODAC counts. Table 1 also shows the percentages of discordant results obtained with the two techniques, using 100 RLU/100 cm^2^ as the benchmark for the ATP values. Of 140 examined surfaces, 120 (85.7%) had concordant results: of these, 119 were compliant with the limits of both ATP (< 100 RLU/100 cm^2^) and RODAC (< 15 CFU/plate) methods and one exceeded both limits. The highest percentage of discordance was observed in samples exceeding 15 CFU/plate; in two of the three surfaces included in this class of microbial contamination, the RODAC counts were just above the limit (TVC = 16 CFU/plate) and the corresponding ATP values were 27 and 63 RLU/cm^2^, respectively.

**Table 1:** Distribution of ATP-bioluminescence values (RLU) in the different RODAC categories (CFU/plate)

ATP-bioluminescence assay	RODAC categories			
	No growth	Expected value TVC ≤ 5 CFU/plate	Acceptable value TVC: 6–15 CFU/plate	Not acceptable TVC > 15 CFU/plate
	N: 74	N: 57	N: 6	N: 3
Geometric mean (CI 95%) RLU/100 cm^2^	29.2 (23.6–36.1)	34.7 (25.6–46.9)	51.7 (13.9–191.5)	74.8 (na)
Median (Range) RLU/100 cm^2^	29 (4–480)	30.5 (4–510)	48 (10–480)	63 (27–233)
Number of samples with discordant results (cut off: 100 RLU/100 cm^2^)	7 (9.5%)	10 (17.5%)	1 (16.7%)	2 (66.6%)

*CI*, Confidence Interval

na: not applicable

The mean RLU values show a rising trend from the RODAC no growth category to the not acceptable category (Table 1). Moreover, the two methods appear consistent in identifying the most contaminated areas, as shown in the comparison between hospitals (Table 2), between Pre- and Post sampling (Table 3) and between sampled surfaces (Table 4). In the comparison between the two hospitals, the number of samples exceeding the expected value of the ISPESL guidelines using the cultural method (RODAC) is greater in hospital A than in hospital B, with statistically significant differences (geometric mean: 2.2 CFU/plate vs 1.5 CFU/plate; *P* = 0.01). This finding is confirmed by the geometric means obtained with the ATP assay (Hospital A: 37.7 RLU/100 cm^2^ vs Hospital B: 29.8 RLU/100 cm^2^), even if in this case the differences are not statistically significant (Table 2).

**Table 2:** Assessment of surface contamination by cultural technique (RODAC) and ATP-bioluminescence assay. Comparison between the operating rooms of Hospital A and Hospital B

	TVC (CFU/plate)					ATP-bioluminescence assay (RLU/100 cm^2^)		
	Total	Hospital A	Hospital B	P	Total	Hospital A	Hospital B	P
Geometric mean	1.8	2.2	1.5	0.01	32.7	37.7	29.8	0.19
(CI 95%)	(1.6–2.0)	(1.7–2.8)	(1.3–1.8)		(27.5–38.9)	(29.6–48.1)	(23.5–37.9)	
Median	0	1	0		29	32	29	
(Range)	(0–41)	(0–41)	(0–16)		(4–510)	(8–356)	(4–510)	

*CI*: Confidence Interval

**Table 3:** Assessment of surface contamination by cultural technique (RODAC) and ATP-bioluminescence assay. Comparison between Pre and Post

	TVC (CFU/plate)			ATP-bioluminescence assay (RLU/100 cm^2^)		
	Pre	Post	P	Pre	Post	P
Geometric mean	2.0	1.6	0.07	41.9	25.4	< 0.01
(CI 95%)	(1.6–2.4)	(1.3–1.9)		(32.3–54.5)	(20.5–31.5)	
Median	1	0		45.5	23.0	
(Range)	(0–16)	(0–41)		(0–510)	(4–468)	

*CI*: Confidence Interval

**Table 4:** Assessment of surface contamination by cultural technique (RODAC) and ATP-bioluminescence assay. Comparison of the different sampled surfaces

Sampled surfaces	TVC (RODAC) (CFU/plate)				ATP-Bioluminescence assay (RLU/100 cm^2^)			
	Geometric mean	CI 95%	Median	Range	Geometric mean	CI 95%	Median	Range
medical anaesthesia trolley	2.1	1.4–3.0	1	0–14	42.2	24.7–72.3	38	6–510
nurse’s computer touch screen	1.1	1.0–1.4	0	0–3	29.9	17.8–50.2	30	4–480
operating table	1.7	1.2–2.5	0	0–13	35.7	21.6–58.9	31	5–468
vitals monitor	1.4	1.1–1.7	0	0–3	29.6	19.7–44.2	32	5–172
anaesthetist’s computer touch screen	1.6	1.1–2.4	0	0–16	27.2	18.2–40.8	26	5–102
surgical lighting	2.6	1.6–4.1	1	0–41	60.5	37.4–98.1	42.5	13–357
Instrument table	2.4	1.7–3.4	1	0–10	18.4	12.4–27.2	13.5	4–106

*CI*: Confidence Interval

With regards the comparison between Pre and Post, the number of samples exceeding the expected value with the RODAC method is greater in the Pre sampling, where the mean value of the microbial counts is also higher (geometric mean: 2.0 CFU/plate vs 1.6 CFU/plate), although the differences are not statistically significant. The same situation can be observed with the ATP assay (geometric mean: 41.9 RLU/100 cm^2^ vs 25.4 RLU/100 cm^2^), with statistically significant differences (*P* < 0.01) (Table 3).

Table 4 shows the geometric means and medians of TVC 36 °C and ATP values obtained for each sampled surface. Among the surfaces sampled with the RODAC method, the most contaminated, though with mean counts within the expected value, are: the scialytic lamp (geometric mean: 2.6 CFU/plate), which in two samples showed values above 15 CFU/plate, the instrument table (geometric mean: 2.4 CFU/plate) and the anaesthesia trolley (geometric mean: 2.1 CFU/plate). The results for the bioluminescence largely confirm the data obtained with the cultural method. The most contaminated points are: the scialytic lamp (60.5 RLU/100 cm^2^) and the anaesthesia trolley (42.2 RLU/100 cm^2^). Despite the concordance of the results obtained with the two methods, analysis with the Pearson test did not reveal a correlation (*r* = 0.169 *P* = 0.046) between the TVC (CFU) and ATP (RLU) values recorded for the same samples.

The internal reference RLU values were calculated in accordance with the previously given definition. 63 RLU/100 cm^2^ (75 percentile of the detected values) and 75 RFU/100 cm^2^ (63 RLU/100 + 20%) resulted as target and alert values, respectively. Using the alert value as the benchmark, 16.5% of samples proved to be above the limit, with similar percentages in both hospital (Table 5). Considering instead the conventional value of 100 RLU/100 cm^2^ as the benchmark, the percentage of samples exceeding this limit decreases to 13.6% (12.7% hospital A; 14.3% hospital B).

**Table 5:** Distribution of samples (%) in the different classes of contamination in accordance with the internally defined benchmarks for ATP-bioluminescence assay

	ATP-bioluminescence assay		
	Expected values	Acceptable value	Alert value
	≤ 63 RLU	64–75 RLU	> 75 RLU
Hospital A	72.7	10.9	16.4
Hospital B	77.4	6.0	16.7
Total	75.5	7.9	16.5

## Discussion

Microbial contamination of the operating theatre surfaces, detected by culture method, was very low. Overall, the mean value of the counts obtained with the RODAC method, expressed as geometric mean, was 1.8 CFU/plate (IC 95%: 1.6–2.0), in line with other reports on the contamination of operating theatre surfaces post-sanitization [20]. Furthermore, bacteria potentially responsible for SSIs, such as *S. aureus*, *Enterobacteriaceae,* Enterococci and *Pseudomonas* spp*.*, were never detected in any of the samples. To the best of our knowledge, there are no other studies in the literature that compared the ATP-bioluminescence test with the microbial counts on surfaces with such a low level of contamination.

No statistical correlation was found between the luminometric (RLU) and microbiological (CFU) data obtained for the same surfaces, confirming that the ATP results cannot be interpreted as indicator of microbial contamination. Other studies found contrasting results in the comparison between luminometric data and microbial counts, reporting no correlation [28–30], or a moderate correlation [16, 18, 27, 31], or a significant correlation [15, 22] between ATP levels and TVC values.

A first reason of these results may be linked to the different areas sampled with RODAC plates and ATP swabs. Possible localized hotspots of contamination can affect the results obtained with both methods and make it difficult to find a correlation. Furthermore, the ATP measurement is correlated to the presence of organic matter not only of microbial origin, but also of blood, protein tissues and skin cells, which frequently contaminates the operating theatre surfaces. In addition, many factors can interfere with the bioluminescence method, raising or lowering the readings: the use of sodium hypochlorite-based disinfectants or the presence of chemical residues [32], the characteristics of the surfaces [33], the microfibre products and plastics used for cleaning and washing [9].

Despite these limitations, considering our results as a whole and not in terms of a comparison between single surfaces, the two methods reached concordant results in order to evaluate the hygienic quality of surfaces: the mean ATP values showed a progressive increase from surfaces with TVC = 0 to surfaces with TVC > 15 CFU/plate and the two methods concurred in identifying the most contaminated areas: Hospital A > Hospital B; Pre samples > Post samples; the scialytic lamp as the most contaminated surface. On the basis of these concordant results, ATP-bioluminescence assay may represent a useful tool for the routine assessment of the effectiveness of cleaning procedures, even in environments where the microbial contamination is low. Furthermore, the ATP measurement provides a result in real time and offers operators immediate feedback of the efficiency of sanitization interventions. The simple awareness that their performance is monitored is in itself sufficient to induce personnel to change behaviours and these changes persist only for the duration of the assessment [14]. Examining the luminometric results obtained in situ and discussing the critical points with the operators can sensitize the staff and improve their adhesion to the standards defined by the sanitization protocols, enhancing the quality of cleaning [29, 34].

The use of the luminometer for the assessment of the hygienic quality of surfaces involves the definition of a benchmark as an alert value [17]. The manufacturer of the 3 M™ Clean-Trace™ NGi Luminometer used in this study, suggests a benchmark value of 250 RLU/100 cm^2^ and recommends that in monitoring high-risk areas at least 90% of the analysed samples should not exceed 250 RLU/100 cm^2^. The same cut-off has been indicated for healthcare environments after best practice cleaning [16, 35]. These values seem too high for the type of surfaces examined in this study. In fact, the mean RLU values obtained for all the samples were 32.7 RLU/100 cm^2^ as a geometric mean and 29.0 RLU/100 cm^2^ as a median, and therefore much lower than the benchmarks proposed. Also the cut-off of 100 RLU/100 cm^2^, conventionally used, could be not very suitable for these environments.

In absence of RLU standard values applicable at a general level, the benchmarks should be established singularly for each environment examined, depending on the instrument used and the levels of contamination detected: in our conditions, with a generally low level of contamination, we calculated the 75 percentile of RLUs as the internal target value (63 RLU/100cm^2^). In accordance with the recommendations of the CDC [9], which suggests taking into account deviations of up to 20% from the established reference values, the alert value was calculated to be 75 RLU/100 cm^2^ (reference value = 63 RLU 100 cm2 + 20%). Using this value as the benchmark, applicable to low contaminated surfaces such as those examined in our study, 16.5% of samples proved to be above the limit, in percentage of 3% higher than the one obtained using the 100 RLU nonspecific benchmark value. The definition of this local standard, more restrictive than the reference values suggested by the manufacturer or commonly used, allowed us to appreciate the global level of contamination of the surfaces, even in the presence of low microbial counts; it also enabled us to recognize the spatial (between operating blocks, operating rooms, and surfaces) and temporal (at different moments of the daily operating session) variations and identify the surfaces that needed greater attention during cleaning and disinfection.

## Conclusions

The ATP-bioluminescence assay cannot substitute the cultural methods in the assessment of environmental microbiological contamination. However, the two methods reached a good level of concordance in order to evaluate the hygienic quality of surfaces. The ATP- bioluminescence method, due to its ease of execution and the immediacy of results, allows you to monitor surfaces more frequently and in greater numbers and can be used as a rapid tool of screening the efficacy of cleaning procedures. In each health facility, in order to interpret the obtained ATP results, internal reference values should be established, on the basis of the device used, the risk level of the relative environment, and the analysis of data collected through spatial and temporal sampling series. When the defined limits are exceeded, the ATP test, by providing a rapid feedback, allow any critical situations to be addressed immediately, as well as increasing the awareness of the operators.

## Abbreviations

ATP: Adenosine triphosphate
CFU: Colony forming units
RLU: Relative light units
RODAC: Replicate organism direct agar contact
spp.: Species
SSI: Surgical site infections
TVC: Total viable count
